# Intersectional Inequalities in Neighbourhood Air Pollution Concentration in England: A Quantitative Analysis of Ecological Data Using Eco-Intersectional Multilevel (EIM) Modelling

**DOI:** 10.1007/s12061-025-09787-8

**Published:** 2026-01-30

**Authors:** Natalie C Bennett, Andrew Bell, Paul Norman, Clare Evans, Remy Veness

**Affiliations:** 1 University of Manchester, Manchester, United Kingdom; 2 University of Sheffield, Sheffield, United Kingdom; 3 University of Leeds, Leeds, United Kingdom; 4 University of Oregon, Eugene, United States; 5 Sheffield Hallam University, Sheffield, United Kingdom

**Keywords:** Inequality, Intersectionality, Environmental justice, Air pollution, EIM, MAIHDA

## Abstract

Air pollution is detrimentally associated with many health outcomes, yet its impacts are not equally distributed. Research consistently finds inequalities by ethnicity, area deprivation and age. However, such inequalities are typically investigated separately, potentially underestimating the extent of differential exposures. We aim to investigate inequalities in NOx concentrations across multiple intersecting neighbourhood characteristics in England simultaneously. We do this using the novel Eco-Intersectional Multilevel (EIM) modelling approach, we define analytic “strata” of neighbourhoods based on sociodemographic characteristics. This enables us to quantify NOx concentration inequalities across community types, simultaneously considering area deprivation, ethnicity, education, rurality and age of residents. We find that neighbourhoods belonging to the “most deprived, high proportion minority ethnic, high education, urban and not ageing” stratum had the highest average NOx concentration. This concentration was five times higher than places with the lowest concentration in the mid deprivation, low proportion minority ethnic, high education, rural and ageing stratum. We find clear and striking inequalities by ethnicity. However, we do not find evidence of inequalities by area deprivation that operate independently of community ethnicity, likely due to the strong relationship between ethnicity and deprivation distributions. This study demonstrates the value of taking an intersectional approach to geographical inequalities.

**Supplementary Information:**

The online version contains supplementary material available at 10.1007/s12061-025-09787-8.

## Highlights

- NOx concentration was analysed across several intersecting place characteristics.

- Eco-Intersectional Multilevel modelling was used to study these inequalities.

- NOx concentration was five times greater in the stratum with the highest versus lowest NOx.

- Large ethnic inequalities were identified after accounting for area deprivation.

**Supplementary Information:**

The online version contains supplementary material available at 10.1007/s12061-025-09787-8.

## Introduction

Globally, air pollution is estimated to cause 6.5 million deaths every year (Fuller et al., 2022) and in England, the UK Health Security Agency estimate the yearly mortality burden to be between 26,000 and 38,000 deaths (Mitsakou et al., 2022). It is also known to worsen a range of health outcomes including asthma and cardiovascular disease. However, exposure to air pollution and its consequent impacts on health are not equally distributed across geography and society. Inequalities in air pollution concentrations have been identified across Western European countries (Fairburn et al., 2019; Samoli et al., 2019) and North America (Hajat et al., 2015; Ard, 2015; Downey & Hawkins, 2008). Typically, it is minoritised and low-income communities that disproportionately bear the burden of exposure. In general, air pollution concentrations have been found to be higher for those from less socio-economically advantaged backgrounds (Hajat et al., 2015; Milojevic et al., 2017) and for minoritised ethnic groups and in areas of greater deprivation (Liu et al., 2021). Furthermore, there are inequalities in the groups who are more likely to contribute a larger share of atmospheric pollution, driven by intersecting processes and histories of power and oppression (Cubells et al., 2024).

Though these inequalities are regularly identified, they are rarely investigated in multiplicative combination. For example, though studies demonstrate greater pollution exposure both in more deprived places and for minoritised ethnic groups, the combination (deprived *and* minoritised) is rarely considered. This practice of studying inequalities in isolation risks misrepresenting and underestimating their true extent. For instance, some communities at the intersection of several ‘at risk’ groups may experience ‘stacked’ disadvantage that cannot be captured in single-axis studies. However, recent work combining ideas from intersectionality theory and geography has begun to emphasise the importance of better understanding this complexity (Ducre, 2018; Malin & Ryder, 2018).

This is the first study to apply the innovative Eco-Intersectional Multilevel (EIM) modelling approach to describe multiple intersecting place-based inequalities using UK data. EIM is a variant of the Multilevel Analysis of Individual Heterogeneity and Discriminatory Accuracy (MAIHDA) approach, developed for ecological data (Alvarez et al., 2022; Alvarez & Evans, 2021). Like MAIHDA, the method facilitates the examination of multiple axes of inequality simultaneously, underpinned by intersectionality theory, while leveraging the methodological advantages of multilevel models for robust predication (Evans et al., 2018; Leckie et al., 2025). Our findings reveal important multiplicative inequalities in pollutant concentration, as well as the importance of ethnicity, over area deprivation, in driving spatial inequalities in pollutants.

### Intersectionality

Intersectionality is a critical theoretical framework that supports understanding how interlocking systems of power produce unique experiences at the intersections of different identities (Crenshaw, 1989; Collins, 1990). The concept relates to systems of both privilege and oppression, and the range of levels at which these systems operate - from individual to structural. A central argument is that an additive conceptualisation of experiences at the intersection of identities is inadequate, serving to erase the unique experiences of multiply marginalised groups (Crenshaw, 1989). Though the theory originally sought to highlight the often-invisibilised experiences of Black women in the US, use of an intersectional lens is now broader and employed to investigate a variety of identity and power combinations.

Much of the qualitative and mixed methods intersectional scholarship focuses on specific subgroups within marginalised or neglected populations. However, quantitative approaches to intersectionality are typically inter-categorical (Mccall, 2005) explorations of inequalities across populations. Quantitative intersectional studies often use interaction terms within regression models in order to examine how inequalities along one dimension (e.g. gender) vary along another (e.g. ethnicity) (Bauer et al., 2021). However, as models specified in this manner quickly become complex and difficult to interpret, the number of interactions included are typically small, and the social categories analysed few. Limitations associated with this kind of modelling have led to the recent development of improved methods, such as intersectional MAIHDA (Multilevel Analysis of Individual Heterogeneity and Discriminatory Accuracy) for investigating intersectional inequalities (Evans et al., 2018; Jones et al., 2016).

Despite the adoption of quantitative intersectional investigation in the social sciences, intersectional approaches have less commonly been applied to quantitative geographical research. This has, for instance, led to calls for a more intersectional approach to geographical health inequalities (Bambra, 2022). The introduction of an intersectional lens to geographical research in particular offers the opportunity to apply a more intersectional perspective to established environmental justice research (Malin & Ryder, 2018).

### Environmental Justice

Broadly speaking, environmental justice (EJ) is concerned with the unequal patterning of environmental resources and quality across marginalised groups. EJ originates from US activism and research, particularly around the siting of industry and hazardous waste processing facilities in close proximity to predominantly Black communities and Native American reservations, and to less socio-economically advantaged communities (Walker, 2012; Lerner, 2012). However, it is argued that interest in EJ in the UK, which gained momentum around the 1990 s, has been largely focused on deprivation, unlike in the US where focus has instead concentrated on race or ethnicity (Mitchell, 2019; Mitchell & Norman, 2012). The beginnings of EJ thought and research in the UK were quite unlike its community-led, activist-driven origins in the US (Walker, 2012). Adoption of the movement was met with the greatest enthusiasm within academic research, charities and Government bodies (such as the Environment Agency) rather than communities themselves (Mitchell, 2019). Walker (2012) notes that the language surrounding EJ became that of ‘environmental inequalities’, rather than ‘injustice’, chiming with the political focus on ‘health inequalities’ (Acheson, 1998) of the New Labour government of the time. Importantly, the idea of ‘injustice’ implies a moral responsibility to act to correct the inequality. How an inequality came to be can make it an injustice, but it can also be considered an injustice if it is harmful, unequally experienced, and requires amelioration.

As research on EJ has progressed, a greater variety of marginalised identities and types of environmental outcomes have been studied. For example, evidence suggests that there are inequalities in exposure to air pollution across socio-economic groups, ethnicity and age in the UK (Barnes et al., 2019; Mitchell & Dorling, 2003; Milojevic et al., 2017; Fecht et al., 2015; Fairburn et al., 2019). Inequalities in exposure to environmental hazards such as air pollution play a role in explaining geographical health inequalities (World Health Organization, 2019; Kaźmierczak, 2018). However, unlike the literature on discriminatory industrial siting, identifying causal processes behind other environmental injustices is arguably more complex.

EJ research, especially focused on air pollution, is usually conducted along a single axis of inequality. Furthermore, these studies are often focused on specific geographically constrained case study sites (Fairburn et al., 2019; Lerner, 2012). While providing valuable depth, the findings of these studies often cannot be extrapolated to wider contexts. Addressing some of these challenges, advances in modelling for intersectionality (MAIHDA) can also be applied to environmental justice questions in an ecological version of the method (EIM).

### Inequality and Air Pollution

As we describe in the introduction, it is often minoritised and low-income groups who come to be exposed to higher concentrations of pollutants. A variety of hypotheses specifically surrounding the development of environmental injustices exist and have been described by Liu (2001) and summarised by Mitchell and Norman (2012). These explanations include: discriminatory siting practices (whereby industry and hazardous facilities are disproportionately located in disadvantaged and marginalised neighbourhoods), risk theory (whereby individual perception of risk varies by social characteristics), neighbourhood transition theory (whereby marginalised groups are forced to live in areas of low environmental quality due to limited income, but become more socially and culturally attractive to particular groups with time), location theory (which emphasises the range of factors taken into account when selecting a residence, of which environmental quality is just one) and land use planning theory (whereby areas with good environmental quality are protected, pushing further degradation onto areas which are already poor quality). However, the processes which determine residential patterns and pollution concentrations are complex and are the result of a combination of geographical processes such as these, as well as social processes, demography and policy.

Urban areas typically have higher average NO_2_/NOx pollution concentrations as a result of high traffic volumes and manufacturing (Prieto et al., 2021; Elliott et al., 2024). This means those living in inner cities are likely to be most exposed. Whilst inner cities are diverse places, other inequalities, processes and power structures mean that certain groups have more ability to relocate or avoid places with high pollution concentrations (such as by busy roads). Research from a US context suggests that residential moves to relatively less polluted places are associated with richer households (Silva et al., 2024). This might suggest that inequality in concentration of NOx could follow similar patterns as inequality in wealth which exists across ethnicity and socio-economic status in England (Cummins, 2024; Gregg & Kanabar, 2025). Conversely, places outside of inner cities have lower pollution concentrations. Evidence suggests that pollution levels are often lower in areas with an older population (Fecht et al., 2015). This is likely due to this population having less need to be near a city as they reach retirement. For example, research suggests that older people may prefer smaller villages and towns over city locations (Mulliner et al., 2020).

In addition to a notable urban disadvantage, inequalities by area deprivation have been observed in England, suggesting more deprived places tend to have higher pollutant concentrations (Gray et al., 2023). As we describe above, there are likely a number of geographical processes behind this (especially relevant are discriminatory siting and land use planning theories). However, a wide variety of forces shape the geographic distribution of area deprivation, beyond just those linked to EJ. For example, it has been argued that the relationship between deprivation and air pollution is more complex than it may appear, being shaped by other processes such as urbanisation and gentrification. This may mean that some of the least deprived areas may also have higher pollution levels (Bailey et al., 2018; Mitchell & Dorling, 2003).

Finally, understanding ethnic inequalities in the UK and the uneven geographic distribution of ethnic groups is important in the context of pollution inequalities. For example, in the US historical ‘redlining’ practices (whereby financial services were withheld from neighbourhoods of colour while provided in predominantly White neighbourhoods) still have a legacy today (Lynch et al., 2021). Though often less overt, discriminatory practices in housing markets are also present across Europe (Auspurg et al., 2019). In England, discriminatory practices surrounding housing access, policy and allocation for people from minority ethnic groups have been described as ‘slippery’ “…in that they can be difficult to precisely evidence and challenge, particularly as they have become embedded and normalised over a long period” p.3201 (Lukes et al., 2019). Changes to housing and immigration policy, the enforcement of everyday borders via requiring landlords to check the immigration status of tenants, the favouring of long term residents in local authority housing allocation, and perceived discrimination by private housing landlords have all impacted the spatial patterning of minority ethnic and migrant housing (Lukes et al., 2019). These historic and contemporary policies and practices, in combination with restricted social mobility and socio-economic inequality (Platt & Zuccotti, 2021), have resulted in disproportionate housing disadvantage faced by minority ethnic groups, and a notable concentration of minority ethnic groups in deprived, often inner-city areas (Lukes et al., 2019; Lees & Hubbard, 2022). Inner city areas themselves have been racialised and stigmatised, being closely tied to ideas of race and ‘segregation’ (Rhodes & Brown, 2019). However, contemporary residential patterns in England show a geographical dispersion of ethnic diversity beyond inner city areas (Catney, 2016).

As policy interventions to tackle pollution and traffic become increasingly common, recognising the potential role of local and national policy on inequality and environmental justice is important. For example, localised air quality policies, particularly when implemented in high-pollution areas, may be a successful strategy to reduce environmental inequalities (Pye et al., 2006). The combination of these simultaneous processes and policies with interlocking and interacting systems of power means that specifically intersectional inequalities are likely to be produced.

### Introducing Eco-Intersectional Multilevel (EIM) Modelling

MAIHDA is well suited to investigating these inequalities. MAIHDA is a modelling procedure in which multilevel models are used to capture the ‘interaction effects’ of many intersections, without the cost to model parsimony encountered in typical single-level models with multi-way interaction terms (Evans et al., 2024b). It does this by conceptualising and generating intersectional groups called ‘strata’ (level two) which individuals (level one) are nested within. Each individual belongs to only one stratum corresponding to the characteristics or identities belonging to that individual, for a set of chosen variables. The number of strata will depend on the number of variables chosen, and the number of categories that each variable is coded into. For example: strata comprised of sex (binary), age (coded into four categories), ethnicity (coded into five) and socio-economic status (coded into four) would produce (2*4*5*4) 160 strata of unique attribute combinations. These strata are then treated as a ‘level’ within a multilevel modelling framework. This facilitates examining multiple, intersecting characteristics important for understanding intersectional inequalities. In addition, MAIHDA benefits from statistical shrinkage (meaning unreliable estimates from small groups are pulled towards the sample mean) (Bell et al., 2019) which is especially beneficial for groups with small sample sizes (Mahendran et al., 2022; Van Dusen et al., 2024; Evans et al., 2024b; Leckie et al., 2025). It has been shown to outperform other methods commonly used for estimating intersectional inequalities (Mahendran et al., 2022; Van Dusen et al., 2024).

EIM modelling is similar to conventional MAIHDA, except that the lower-level unit of analysis is geographic areas, rather than individuals. These geographic areas (e.g. neighbourhoods) are clustered by strata (otherwise conceptualised as analytic ‘community types’) typically defined using a combination of aggregate sociodemographic characteristics. Such a model allows for the consideration of intersectional inequalities in variables such as pollution that are measured at the ecological, rather than individual, scale.

Recent research from the US using an EIM approach has revealed stark intersectional environmental inequalities (Alvarez et al., 2022; Alvarez & Evans, 2021). The authors find substantial differences in air pollutant concentrations in the USA by key census tract characteristics including ethnicity, education, household income and urbanicity. The studies reveal that particular types of places, particularly those defined by multiply marginalised characteristics, are more likely to have much higher pollutant concentrations. EIM analysis enabled them to test the generalisability of the findings in prior EJ scholarship – which was often case study based and focused on individual communities – that multiply marginalised communities faced greater risk of harms.

The application of individual-level MAIHDA has begun to provide insight into who contributes most to atmospheric emissions (Cubells et al., 2024). However, despite a large body of research on single axes of inequality in air pollution, evidence of intersectional inequalities in the UK remains scant. Additionally, we argue that much of the research on air pollution in England remains focused on area deprivation, often to the detriment of a more nuanced and comprehensive understanding of the unequal patterning of exposure to this environmental hazard. Improved knowledge of the socio-spatial patterning of air pollution could facilitate the development of environmental policies better tailored to the reduction of environmental inequalities, as well as overall environmental improvement. Therefore, in this paper we aim to apply novel EIM modelling methods in order to describe neighbourhood level eco-intersectional inequalities in estimated NOx concentration across several place characteristics.

## Methods

### Data

In this analysis, we employ Lower Super Output Area (LSOA) administrative units to capture geographical units which are approximate to neighbourhoods. There are 33,755 LSOAs in England and there are between 1,000 and 3,000 people per LSOA (Office for National Statistics, 2024). We focus on England, in part due to a lack of comparability of some area-based measures including area deprivation across UK countries, as well as inconsistencies in emissions regulations and policies across countries.

### Dependent Variable: NOx

We use average annual ambient concentration of oxides of nitrogen (NOx) as our dependent variable of interest. NOx includes both nitrogen dioxide (NO_2_, which is released into the atmosphere when burning fuel at high temperatures) and nitrous oxide (NO), which typically co-occur. In the UK, around one third of NOx emissions originate from road-traffic, while the remaining two thirds come from a mix of other transport types, manufacturing, machinery, other combustion and combustion industries (Elliott et al., 2024). The Air Quality Standards Regulations (HM Government, 2010) specify that annual concentrations of NO_2_ should not exceed 40 µg m^− 3^ (though, being a combined group of gasses, no limit specific to NOx is available).

**Fig. 1:**
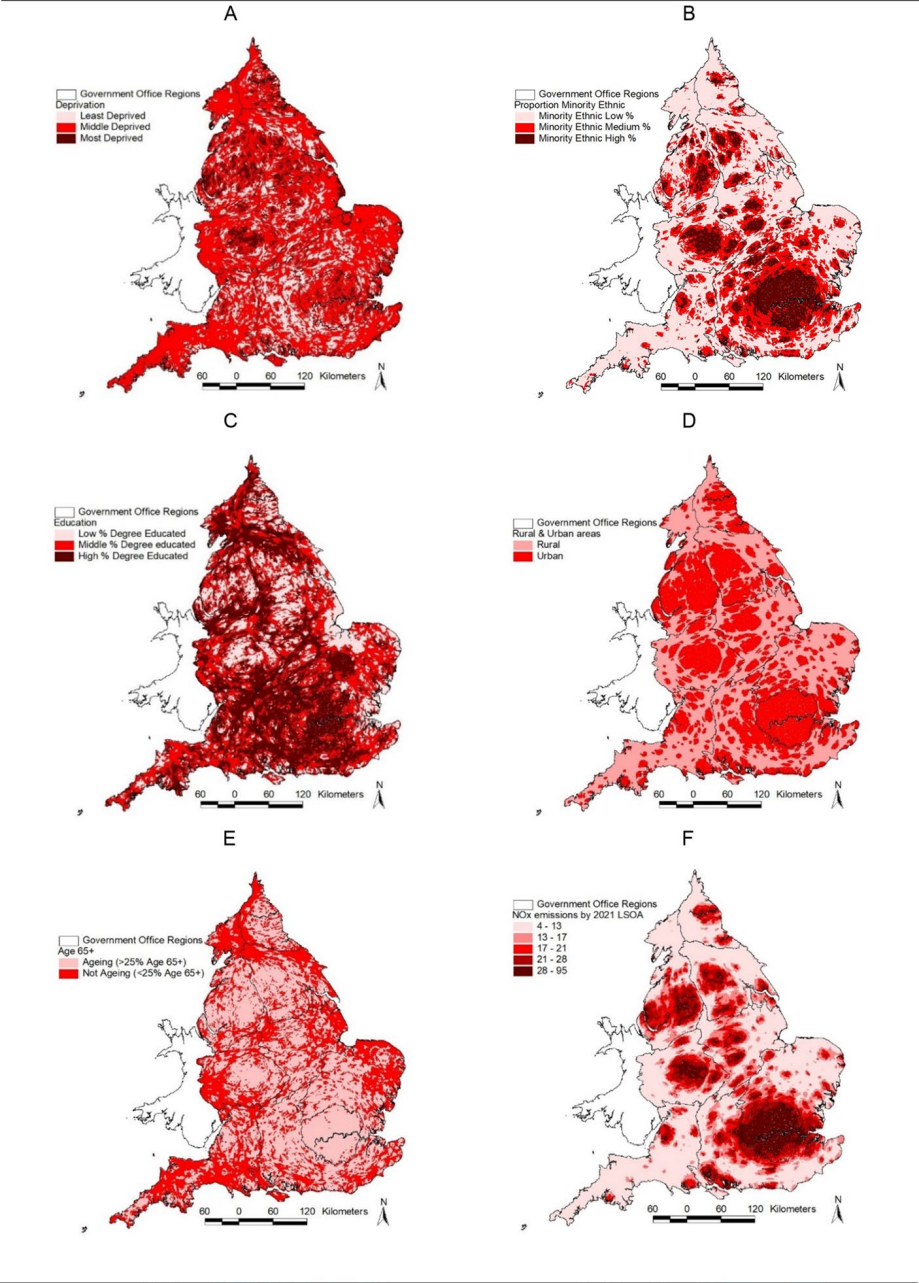
NOx (µg m^− 3^) and five stratum variables presented using the 2021 Lower Super Output Area (LSOA) geography. The LSOAs are presented as a cartogram based on the square root of each LSOA’s area. In the cartogram, the size of urban and rural areas are scaled up and down respectively (Norman et al., 2024)

The NOx data used in this paper are freely available online from the Department for Environment, Food & Rural Affairs UK Air Information Resource (Department for Environment Food & Rural Affairs [Defra], 2024) and are provided as annual mean µg m^− 3^ estimates. More detail on the NOx data and preparation process is provided in the appendix. We use 2019 data on NOx background emissions as this is the final edition of the data prior to the COVID-19 pandemic which substantially impacted emissions data and its social patterning due to extended periods of restrictions to population mobility. We visualise these data, along with the other variables used to produce our strata in Fig. 1, and descriptive statistics are found in Table 1.

### Stratum Variable Sources

Data on age, ethnicity and education are sourced from the 2021 Census (Nomis, 2024). This is the most recent edition of the census and the most temporally proximal to the 2019 NOx data. Neighbourhood-level demographics change slowly and are unlikely to be significantly different had they been measured three years previously. Regardless, we conduct the present analysis as a theoretically informed descriptive exercise and make no causal claims relating to how the estimated inequalities came to be.

The most up to date indicator of urbanicity available at the time of analysis was from the 2011 Census and is primarily based on population patterns. These data were available only at 2011 LSOA boundaries and were recalculated to the most up-to-date LSOA boundaries (details available in the appendix). Education, social mobility and racism affect the patterning of residential choices, including whether a person resides in an urban or rural location. We therefore consider rural-urban as a stratum defining variable, as another potential axis of inequality (we also consider models excluding the rural-urban variable).

Finally, our deprivation data are from the most recent edition of the (2019) Index of Multiple Deprivation available at the time of analysis (Department for Communities and Local Government [Dclg], 2019). These data are also produced at the 2011 LSOA scale boundaries and so were rescaled to the 2021 LSOA boundaries employed in the census data (see appendix).

### Stratum Variable Coding

Stratum variables must be categorical to facilitate stratum construction and the examination of interaction effects (a non-parametric concept). This requires compromise between retaining model complexity and diversity in variables on the one hand, and on the other maintaining large enough strata to allow meaningful analysis (Evans et al., 2024a). We therefore categorised continuous variables into ordinal quantiles in order to retain some variability across LSOAs. The strata are derived from combinations of categories of the following variables:

- The Index of Multiple Deprivation (IMD): a composite measure which includes a wide range of data about local places, with a view of assigning a relative score of deprivation to small areas, so that they can be compared. IMD is a weighted score of seven domains: Income, Deprivation, Education, Skills and Training, Health and Disability, Crime, Barriers to Housing and Services, and Living Environment. We reclassify these into three categories: the 20% most deprived, the middle 60% and the 20% least deprived, in line with common practice in health research (Department of Health and Social Care, 2024).
- The proportion of the population who are minority ethnic (defined excluding White British and all other White backgrounds). We produce tertiles of the proportion of the population of LSOAs which are minority ethnic from low to high (1–3).
- The proportion of the population in each LSOA that are educated to degree level (or equivalent) or above, categorised into tertiles low to high (1–3).
- A binary indicator of the rural-urban classification. Further information on the calculation of this indicator is included in the appendix.
- A binary indicator of whether LSOAs have over 25% of the population aged 65 and above (broadly aligning with retirement age).

Descriptive tables of each of these variables across LSOAs in England are provided in Table 1. The combination of the categories of each of these variables provides a code with five digits, ordered as above. For example, code 21301 represents LSOAs within the mid-deprivation (2), low proportion minority ethnic (1), high education (3), rural (0) and ageing strata (1).

### Analysis Methods

We employ EIM methods, whereby LSOAs are nested within intersectional strata. As described above, EIM mirrors the MAIHDA analysis technique (Evans et al., 2018) except for in its lowest unit of analysis, which in EIM are areas rather than individuals. As with any ecological analysis, it is important to be cognisant of the level at which our data are measured and therefore, the level at which inferences can be made in order to avoid the ecological fallacy (Piantadosi et al., 1988; Robinson, 2009). In this study, we aim to describe inequalities in pollution across neighbourhood level characteristics, not across people within them. Therefore, the analysis and results relate to neighbourhood level inequalities. The patterns we identify at the neighbourhood level should not be assumed to apply to individuals. Nevertheless, since many social processes that produce pollution operate at the level of communities, it is appropriate to evaluate their unequal patterning at the community level.

We specified two primary models: in Models 1a and 1b we define strata using deprivation, minority ethnicity, education, and age, while in Models 2a and 2b we additionally define strata by rural/urban location. Models 1a and 2a are null (or ‘empty’) versions of the multilevel models, with only the NOx dependent variable and the stratum-level structure. In other words, only an intercept (representing the predicted precision-weighted grand mean across all strata) is included in the fixed part of the model, while stratum residuals capture the difference between a given stratum’s NOx prediction and that global average.

$$\:{y}_{ij}={\beta\:}_{0}+{u}_{j}+{e}_{ij}$$

$$\:{u}_{j}\:\sim\:N(0,{\sigma\:}_{u}^{2})$$

$$\:{e}_{ij}\:\sim\:N(0,{\sigma\:}_{e}^{2})$$

In these equations, $\:{y}_{ij}$ represents the NOx concentration of a given LSOA *i* in a given stratum *j*. $\:{\beta\:}_{0}$ represents the intercept, the stratum-level residual by $\:{u}_{j}$, and the LSOA-level residual for LSOA *i* in a given stratum *j* is represented by $\:{e}_{ij}$. The residuals for both LSOAs and strata were assumed to be normally distributed with a mean of 0 and a between-LSOA/within-stratum variance of $\:{\sigma\:}_{e}^{2}$ and a between stratum variance of $\:{\sigma\:}_{u}^{2}$. This model tells us how much of the total variance in NOx among LSOAs can be explained by patterns of inequality at the stratum-level.

In Models 1b and 2b (“main effects” models), the LSOA demographic variables used to define the strata are included. We conceptualise this model as now additionally examining ‘additive effects’, meaning we now include stratum-defining variables in the fixed part of the model. In this model, the stratum-level residuals $\:{u}_{j}$ can be interpreted as the difference between the total predicted value for a particular stratum and the stratum value that would be expected based only on the additive variable effects in the fixed part of the model – in other words, “interaction effects”. Departures in predicted value in either magnitude or direction from what we would expect from the general way these variables behave in our model suggest something unique may be happening in that particular stratum. In line with intersectional thinking, we therefore take any positive or negative values of the stratum-level residual variance as indication of the presence of “interaction effects” unique to each stratum. This allows us to consider whether particular strata are associated with particular (dis)advantage, that is greater/lower concentrations of NOx than expected given that specific stratum’s combined additive (dis)advantages.

Two additional valuable statistics, the Variance Partition Coefficient (VPC) and Proportional Change in Variance (PCV), are calculated as part of the MAIHDA/EIM modelling process. The VPC (using statistics from the null model) is calculated as:

$$\:VPC=\frac{{\sigma\:}_{u}^{2}}{{\sigma\:}_{u}^{2}+{\sigma\:}_{e}^{2}}$$

This describes the proportion of variance in the outcome which can be attributed to the between-stratum-level (level 2). In other words, it is a global measure of inequalities between the strata standardized against the amount of level 1 variation. This statistic is also calculated in the same manner using the ‘main effects’ model statistics, though with a different interpretation; VPC in Models 1b and 2b describes residual inequalities between strata that we attribute to interaction effects.

The second statistic, the PCV, describes the change in stratum-level variance between the null and additive models:

$$\:PCV\:=\:\frac{{\sigma\:}_{u,\:Model1a}^{2}\:-\:{\sigma\:}_{u,Model1b}^{2}\:\:}{{\sigma\:}_{u,Model1a}^{2}}$$

The PCV statistic describes the proportion of the total between-stratum variance in the null model ($\:{\sigma\:}_{u,Model1a}^{2}$) which is accounted for by the additive main effects. The PCV statistic thus provides a measure of the extent to which between-stratum inequalities are additively patterned (and therefore more consistent or predictable) as opposed to requiring conceptualisation of ‘interaction effects’ in order to describe unexpected deviations from those additive inequality patterns. A tutorial with example code is available for regular MAIHDA (identical to our approach except for the level one units being individuals) - see: (Evans et al., 2024b).

### Model Specification

We run the first two models (both the ‘null’ and ‘main effects’ models, 1a and 1b) without including the rural-urban indicator (in this model, there are therefore only 52 strata). However, rural-urban differences are likely given that NOx is predominantly emitted from motor vehicles and is an important aspect of inequalities. We therefore subsequently run two further models which include the indicator (models 2a and 2b) in order to examine these differences and the extent to which this explains the patterns we identify in models 1a and 1b.

In analyses 1a and 1b, 33,755 LSOAs in England are nested within 54 strata, 52 of which contain at least one LSOA. In the second set of models (including the rural-urban indicator) (Models 2a and 2b), there are 108 strata, 94 of which contain at least one LSOA; the remaining 14 are empty (see appendix Table A1 for a list of these empty strata).

All models were run in StataMP 18 (StataCorp., 2023) and use maximum likelihood estimation. Sensitivity analyses pertaining to the atypical relationship between public transport and deprivation in London, the IMD living environment and education domains, controls for population density (including an analysis of rural areas only) and spatial autocorrelation are presented in the appendix (see tables A5-A10).

## Results

Table 1 presents the descriptive statistics, including the mean NOx concentration across LSOAs at 21.29 µg m^− 3^ (ranging from 3.62 µg m^− 3^ to 95.42 µg m^− 3^). Other demographic variables included in the model also vary widely across LSOAs. Results from models 1a-2b are presented in Table 2.

**Table 1:** Descriptive statistics

Variable	Mean	SD	Median	Min	Max	Range/Categories in strata
NOx modelled concentration	21.29	10.87	19.09	3.62	95.42	3.62–95.42
Index of Multiple Deprivation score	21.66	15.27	17.67	0.54	92.74	Least deprived 20% (1) [*n* = 6,751 (%20.00)] Middle deprived 60% (2) [20,254 (%60.00)] Most deprived 20% (3) [6,750 (%20.00)]
% minority ethnic	17.84	20.48	8.50	0.00	99.20	(tertiles) Low (1), medium (2), high (3) Each [n = 11,252 (%33.33)]
% educated to L4 and over	33.63	12.73	31.46	9.05	87.22	(tertiles) Low (1), medium (2), high (3) Each [n = 11,252 (%33.33)]
Rural-Urban	0.83	0.38	1	0	1	(binary) Rural (0) [*n* = 5,757 (%17.06)] Urban (1) [*n* = 27,998 (%82.94)]
% aged 65 and over	18.92	8.55	18.28	0.07	65.54	(25% threshold) Not ageing (0) [*n* = 25,678 (%76.07)] Ageing (1) [*n* = 8,077 (%23.93)]

**Table 2:** Full model results

	1a	1b	2a	2b	3
IMD19 (Ref: least deprived)					
Mid deprived		0.59		0.49	0.45
95% confidence interval		[−1.48 2.66]		[−0.91 1.89]	[−0.87 1.78]
Most deprived		2.65*		1.65	1.43
		[0.34 4.96]		[−0.09 3.39]	[−0.23 3.09]
Tertiles of % population minority ethnic (Ref: low % minority ethnic)					
Medium % minority ethnic		4.87***		3.67***	3.33**
		[2.74 6.99]		[2.24 5.09]	[1.30 5.37]
High % minority ethnic		13.16***		10.68***	6.94***
		[10.94 15.38]		[9.03 12.32]	[4.11 9.76]
Tertiles of % population L4 educated (Ref: low education)					
Medium education		−0.61		−0.01	−0.03
		[−2.74 1.51]		[−1.51 1.49]	[−1.45 1.40]
High education		0.76		0.37	0.38
		[−1.47 3.00]		[−1.21 1.96]	[−1.12 1.89]
Rural-Urban Classification (Ref: rural)		-			
Urban		-		5.07***	3.80***
		-		[3.77 6.37]	[1.92 5.68]
Interaction term (Ref: low % minority ethnic * rural)					
Medium % minority ethnic * urban		-		-	0.70
		-		-	[−2.02 3.42]
High % minority ethnic * urban		-		-	5.34**
		-		-	[1.96 8.72]
Binary indicator of 25% of the population aged 65+ (Ref: not ageing)					
Ageing		−2.89**		−1.84**	−2.05**
		[−4.69 −1.10]		[−3.10 −0.58]	[−3.26 −0.85]
Intercept	19.57***	14.16***	17.58***	10.75***	11.59***
	[17.77 21.37]	[11.68 16.64]	[16.31 18.84]	[8.93 12.58]	[9.68 13.51]
Between-stratum variance	42.06	9.37	35.66	7.40	6.57
Within-stratum variance	51.67	51.67	49.20	49.21	49.20
Number of observations	33,755	33,755	33,755	33,755	33,755
Number of strata	52	52	94	94	94
AIC	229221.2	229162.6	227693.2	227584.4	227578.7
VPC	44.87%	15.35%	42.02%	13.07%	11.78%
PCV	-	77.72%	-	79.26%	81.58%
*p < 0.05, **p < 0.01, ***p < 0.001					

### Null Models

The VPC from the null model (with the strata structure produced excluding the rural-urban indicator) (Model 1a) was 44.87%, meaning that there is a very high degree of clustering at the stratum level; a substantial proportion of the variance in NOx concentration across LSOAs is attributable to between-strata differences. The VPC from the null model with the strata structure produced including the rural-urban indicator (Model 2a) is similar: 42.02%. These values show that notable inequalities in NOx concentration exist in England across our strata.

### Main Effects Models

Results from the main effects models (Models 1b and 2b) show the extent of the between-stratum variance that is explained once the main (additive) effects are included in the model (once the stratum-defining variables are included in the model). Comparing model 1b and 2b, the results are highly similar. Coefficient estimates of other variables are generally larger in the model excluding rural-urban. However, they remain similar in significance and direction. Due to this similarity, and our interest in urban and rural trends as part of the social patterning of NOx inequality, we focus henceforth on the models including the rural-urban classification.

In the main effects model, (Table 2, Model 2b) where we can examine additive patterns, we find that areas with a medium to high proportion of the population being minority ethnic, and urban areas were significantly more likely to have higher NOx concentrations. Further, we find that the estimated difference between urban and rural areas, controlling for the other variables in the model, is approximately half of the difference between the highest and lowest ethnicity tertiles. Areas which are ageing have, on average, lower NOx concentrations.

**Fig. 2:**
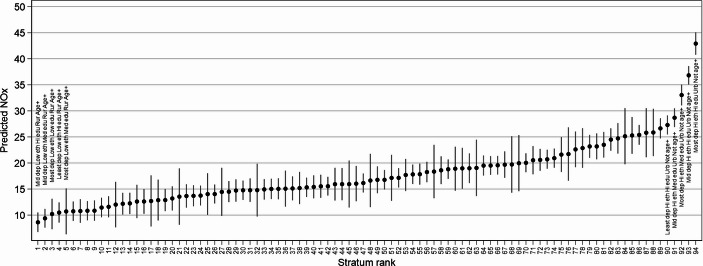
Expected NOx concentration for each stratum (Model 2b)

The VPC of Model 2b reduces to 13.07%, with a PCV of 79.26% (including the rural-urban variable). This suggests that, while additive patterns do explain much of the between-strata variability (inequality), a substantial proportion remains, suggesting interaction effects are needed in order to adequately characterise inequality patterns. This is a greater amount of multiplicative variance than is commonly found in MAIHDA analyses (where PCVs of ~ 85%−95% are more common (Evans et al., 2024a). The presence of interaction effects suggests some strata are deviating from the ‘typical’ inequality patterns described by the additive parameters – either in magnitude or direction. Figure 2 presents a caterpillar plot of the predicted NOx values and 95% confidence intervals (CIs) from Model 2b for each of the strata, ranked. We produce an additional table to describe the stratum characteristics of the top and bottom ten estimated values in appendix Table A2.

Strikingly, the 10 strata with the highest estimated NOx concentrations are all in the highest tertile of the proportion of the population who are minority ethnic, and all but one are urban (Table A2). Education and deprivation vary, while the strata are mostly not ageing. Of the 10 strata with the lowest estimated NOx concentrations, all are low proportion minority ethnic and all are rural. Education, IMD and age all vary, though the lowest six strata are all classified as ageing.

Given the prominence of ethnicity and urbanity in these findings, we additionally wanted to investigate whether there was a systematic, two-way interaction between these variables to examine the degree to which inequalities align with these descriptors specifically. Building on Model 2b, additionally including an interaction term between these two variables in a separate model (Model 3) revealed a statistically significant interaction between the high proportion minority ethnic and urban category (see Table 2, visualisation of the interaction presented in appendix Figure A1). Predicted NOx for the stratum with the highest concentration (most deprived, high proportion minority ethnic, high education, urban, not ageing) was 42.9 µg m^− 3^, compared to 8.6 µg m^− 3^ for the stratum with the lowest concentration (mid deprivation, low proportion minority ethnic, high education, rural, ageing). The NOx concentration is therefore five times higher in the stratum with the highest concentration, compared to that of the lowest. It should also be noted that the Air Quality Standards Regulations state that annual NO_2_ concentrations must not be greater than 40 µg m^− 3^ (HM Government, 2010).

**Fig. 3:**
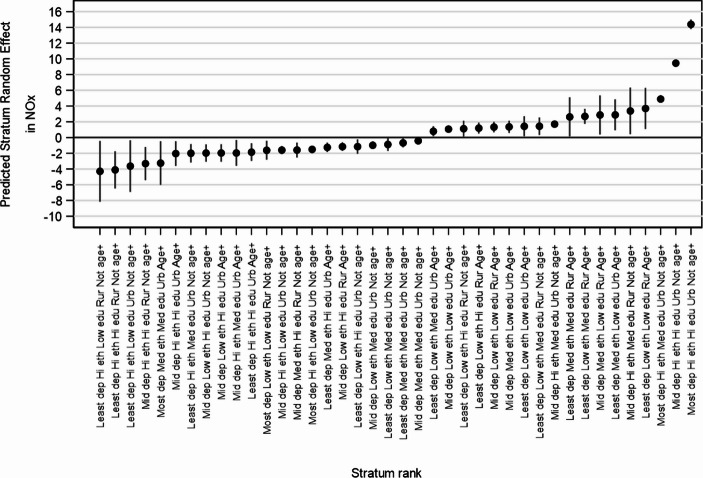
strata with statistically significant interaction effects (Model 2b)

Though additive effects are important to understand, we must also consider the interactive effects by examining the model 2b residuals. We plot the 40 strata with statistically significant interaction effects in Fig. 3 (a table of characteristics and exact estimates for each of these strata is provided in the appendix in Table A3). Strata which are identified as having statistically significant interaction effects can be thought of as having NOx concentrations which are different to what we would have expected from the additive data, though these values should also be interpreted in the context of additive patterns. The quantity of strata with statistically significant residuals and their estimates suggests that interactive effects play an important role in understanding environmental inequalities in NOx concentration. For example, stratum 33310 (most deprived, high ethnicity, high education, urban, not ageing) has the largest mean NOx value (45.07 µg m^− 3^), being at the very end of the spiked tail of Fig. 2, but it also has a large interaction effect (Fig. 3). This is true for several of the strata at the most exposed end of the distribution. This suggests that there some strata which break away from additive patterns, demonstrating synergistic interaction effects.

## Discussion

Using EIM methods for the first time in a European context, we find inequalities in LSOA-level NOx concentration of substantial magnitude. We find that LSOAs within the highest ranked strata (most deprived, high proportion minority ethnic, high education, urban, not ageing) had an average NOx concentration five times higher than the lowest ranked strata (mid deprivation, low proportion minority ethnic, high education, rural, ageing) and 3.3 times higher than the lowest ranked urban strata (mid deprivation, low proportion minority ethnic, high education, ageing). Over 42% of the variance in NOx was attributed to the stratum-level (Model 2a), suggesting that NOx pollution levels at the community level are very unequally experienced across community types, defined by sociodemographic characteristics and urban/rural classification. In particular, we find notably elevated average NOx concentrations for three strata, all of which were of high proportion minority ethnic backgrounds, urban and not ageing.

Including the additive effects of the axes of inequalities captured by the strata did not completely explain the stratum-level variance (over 20% of the stratum variance remained), suggesting interactive effects – where some strata “stand out” as having unusually higher or lower NOx pollution levels than expected. Notably, the three strata with the highest average NOx concentrations – high proportion minority ethnic, urban, not ageing- varying only on education (mid-to high) and deprivation (mid to most deprived) – all had significant positive interactive effects. The combination of these three characteristics (ethnicity, urban and age) in particular seems to be associated with higher pollution levels than might be expected given their already substantial additive effects.

Previous research demonstrates higher concentrations of NO_2_ (Pye et al., 2006; Fecht et al., 2015; Horton et al., 2023) and NOx (Briggs et al., 2008; Gray et al., 2023) in more deprived compared with less deprived areas in the UK. However, our analysis suggests no clear pattern of NOx concentrations by area deprivation when ethnicity and urbanicity are included in the analysis. We note that deprivation does matter the inequalities we observe in NOx, but it does so in combination with ethnicity and urbanicity. Though we find LSOAs in the least deprived 20% are unlikely to be in either the 10 areas of highest or lowest NOx concentrations, analyses of strata with statistically significant interactive effects reveal no discernible patterns. This is perhaps indicative of the more complex relationship between air pollution and area deprivation as a result of other processes like gentrification as suggested by Bailey and colleagues (Bailey et al., 2018).With regards to ethnic inequalities, recent research has begun to provide evidence for inequalities by ethnicity in the UK (Abed Al Ahad et al., 2022). Our paper joins a growing body of evidence from Europe indicating that ethnic inequalities in air pollution exist (Hoy et al., 2024) even after accounting for socio-economic indicators (Ehler et al., 2023; König, 2024) and these dwarf the estimated inequalities by area-level deprivation.

Our findings also align with those of previous studies suggesting that air pollution concentration (in the form of NO_2_ or NOx) follows an age gradient and is typically the lowest in ageing areas (Horton et al., 2023; Mitchell & Dorling, 2003; Barnes et al., 2019). This may be due to a preference of retirees to move out of cities to more suburban locations (Mulliner et al., 2020). Furthermore, our finding of higher average NOx concentrations in urban areas is also mirrored in research on other air pollution types, such as PM2.5 (Milojevic et al., 2017) and in studies assessing NO_2_ and NOx separately (Briggs et al., 2008). Finally, there is relatively limited existing evidence on educational inequalities in NOx or other similar air pollution concentrations. However, Briggs et al. (2008) conclude that education has a weak association with NO_2_ concentration.

As outlined in the literature review, a range of hypotheses surrounding environmental justice exist. These include discriminatory siting, risk theory, neighbourhood transition theory, location theory, and land use planning theory (Liu, 2001; Mitchell & Norman, 2012). These likely combine with other social processes to produce the inequalities we observe. We further expect wider processes of marginalisation, as well as both the legacy of past discriminatory practices and present structural discrimination to influence inequalities.

We find neighbourhoods with more people from minority ethnic groups typically have higher NOx concentrations. Some possible explanations for this pattern pertain to migration and settlement by people from outside of the UK to towns with international transport hubs and/or industrial centres for work, along with more complex residential processes (Shankley & Finney, 2020; Phillips & Harrison, 2010). Neighbourhood transition theory and the persistence of discriminatory residential processes may then explain the continued residence of minority ethnic groups in areas of relatively high pollution, despite the long-since-disappeared draw of employment in manufacturing industries. Discriminatory residential processes can be difficult to evidence in the UK (Shankley & Finney, 2020). However, based on the evidence available on housing and discrimination in the UK, it is likely that racist practices at the local level and policy influence the residential choices made by people from minority ethnic groups (Shankley & Finney, 2020; Lukes et al., 2019; Lees & Hubbard, 2022). Furthermore, inequities in education and social mobility also likely serve to constrain residential choice of minority ethnic groups (Platt & Zuccotti, 2021; Cummins, 2024; Silva et al., 2024). This might explain why we see high proportion minority ethnic urban areas which are mid- to highly educated featuring as areas with some of the highest NOx concentrations. Having examined the geographical locations of LSOAs within these strata, an alternative explanation may be that many (though not all) of the LSOAs in the strata with the highest concentration are located in university towns. These may be more youthful and ethnically diverse places due to the students who move there to study being largely well educated and, in some cases, more likely to be living in more affordable (and perhaps, deprived) places. However, further examination found that the three most polluted strata had only slightly higher rates of student populations than average (see appendix) suggesting that is not driving these patterns.

However, we should not conclude that a lack of a single clear unjust mechanism for creating the inequality in NOx concentrations we observe implies no environmental injustice. Importantly, research suggests that it is commonly neighbourhoods contributing the least to air pollution which are most exposed (Mitchell & Dorling, 2003; Fairburn et al., 2019). Furthermore, under a rights-based approach to EJ, we should all have an equal right to breathe clean air. While explanations for the observed patterns of inequality are certainly important, our present analysis aims to identify and quantify the existence of such patterns, with the intention of ameliorating them in the future. In effect, the existence of these inequalities is a matter of social concern regardless of how they were produced. Therefore, despite a lack of clarity on the mechanisms themselves producing this injustice, that this stark inequality exists demonstrates the importance of an EJ lens.

Interventions in high pollution areas may be an effective way to tackle this inequality (Pye et al., 2006). Evidence from London suggests that the implementation of ‘low traffic neighbourhoods’ (area specific infrastructure-based interventions to reduce or remove motor vehicle traffic) have largely been equitable, being more commonly introduced in low car ownership areas and more likely in more deprived areas (Aldred et al., 2021) and have successfully reduced NO_2_ concentrations in target areas in London (Yang et al., 2022). Further research is needed to establish whether traffic interventions outside of London are similarly equitably sited and impactful to pollution concentrations.

## Limitations and Future Research

Whilst we are able to provide detailed descriptive information on intersectional environmental inequalities, the data and methods employed here do not identify causal processes underlying these inequalities. For example, we cannot say the extent to which structural racism and its implications for social mobility constrain residential choice for people from minority ethnic groups. We are therefore unable to make claims about whether the *processes* that produced these inequity patterns, whereby areas with more people from minority ethnic groups disproportionately have higher average annual NOx concentration, are unjust. However, this evidence of a striking inequity in environmental hazards exposure carries an ethical obligation to act to address the inequity, and failing to do so would constitute an injustice.

A further limitation is that our choice of analytical categories to capture our strata likely does not reveal the true extent of inequalities. For example, our categorisation of ethnicity into a binary (tertiles of proportion minority ethnic) is not ideal, particularly acknowledging that the five largest ethnic groups in England (Indian, Bangladeshi, Pakistani, Caribbean and African) all have different patterns of residential spatial distribution and clustering (Catney & Simpson, 2010; Tonne et al., 2018). However, operationalising ethnicity in this way allows us to balance capturing possible underlying mechanisms (such as those related to broad migration histories, as well as racism) with the demands on the models and the interpretability of the results – a compromise between too fine categories (that are too small to identify any meaningful inequalities) and too coarse (where important within-category inequalities would be missed).

In addition, we acknowledge that spatial autocorrelation is an important potential issue. The models we present do not account for the fact that some LSOAs and strata are closer together than others. Accounting for spatial autocorrelation in EIM presents a challenge, especially in combination with random effects where these are spatially defined. In a sensitivity analysis accounting for a spatially autocorrelated error structure (see appendix Table A10) we find the main results to be broadly consistent with the main results of model 2b. However, reflecting the aforementioned challenges pertaining to random effects, we find VPCs of zero in both models. We do not account for spatial clustering in our primary models, since it is likely that the processes driving the inequalities we observe would to some extent be captured by this. Controlling for this would therefore be undesirable. However, future work could begin to examine the spatial patterning of the inequalities we find and the potential spillover effects that may drive these results.

As the implementation of policies aimed at environmental improvement increases in the UK, future evaluation studies may benefit from the use of MAIHDA or EIM in order to better understand their effects on environmental inequality and injustice. Existing research suggests widening inequalities in air pollution exposure by deprivation (Mitchell et al., 2015; Horton et al., 2023) despite overall air pollution improvements. EIM analyses could facilitate a more nuanced understanding, beyond area deprivation alone, of who benefits from air pollution policies. Research also suggests that exposure to air pollution may exacerbate the detrimental association between area deprivation and health (Brunt et al., 2017). MAIHDA could help provide more detailed knowledge of the potential varying (un)equal impacts of air pollution policies and the disproportionate burden of poor health borne by different groups living in deprived areas.

EIM also allows for investigations beyond typical axes of inequality studied in health research, into more geographical applications of intersectionality theory which are currently lacking (Bambra, 2022). For example, characteristics of place such as its industrial heritage can be important in understanding wider geographical time trends (see (Sinnett & Norman, 2024) for an example). These characteristics could be incorporated as an element of the strata in future research for example to better understand North-South health inequalities in England.

## Conclusion

Existing evidence suggests inequalities in area-level air pollution exist across many important social dimensions. However, research understanding how these interact and whether they do so in an important and meaningful way is lacking, particularly in a UK context. Our analysis of intersectional ecological inequalities using EIM modelling reveals large inequalities between the strata with the highest and lowest NOx concentrations, and stark patterns of inequality, particularly by the ethnic composition of neighbourhoods. We find that the stratum with the highest NOx concentration has an average concentration five times higher average than that which has the lowest. Further, our analysis of interactive effects suggests that additive contributions of the social dimensions comprising the strata analysed are not sufficient to explain the NOx concentration inequalities observed, with younger, high proportion minority ethnic, urban areas standing out as having exceptionally high NOx pollution concentrations. Understanding which multiply marginalised communities are disproportionately exposed to environmental hazards may help to explain the unequal health burdens these groups often also bear. As air pollution interventions become increasingly common in the UK and Europe more widely, EIM offers a potential analysis method for future works aiming to understand the intersectional, and potentially unequal impacts of environmental policy.

## Supplementary Information

Below is the link to the electronic supplementary material.

ESM1 (DOCX.99.1 KB)

## Data Availability

The data used in the manuscript are all freely available to download from the original sources provided.
