# Supplementary material for: Intersectional Inequalities in Neighbourhood Air Pollution Concentration in England: A Quantitative Analysis of Ecological Data Using Eco-Intersectional Multilevel (EIM) Modelling
Source: Appl Spat Anal Policy. 2026 Jan 30;19(1):23. doi: 10.1007/s12061-025-09787-8 (PMC12858503; doi:10.1007/s12061-025-09787-8)
Supplement: Supplementary file 1 — (DOCX.99.1 KB) [file 12061_2025_9787_MOESM1_ESM.docx]

**Appendix**

1. **Additional methods details**

**1a. Reclassifying gridded NOx to 2021 LSOA census boundaries**

NOx is a model-derived estimate and is informed by UK National Atmospheric Emissions Inventory NOx estimates. Measurements are taken at both roadside locations and as background emissions and combined to produce average annual ambient NOx µg m^-3^ dataset used in this study. Greater detail on the modelling process is provided in DEFRA’s technical report (Pugsley et al., 2023). DEFRA release these data as a gridded, 1km resolution product. It was therefore necessary to re-scale these data to the LSOA level for the purposes of our analyses of neighbourhood-level inequalities. We follow a similar process to that implemented by Barnes and colleagues (Barnes et al., 2019).

We calculated NOx for each LSOA using DEFRA’s modelled pollution dataset (Department for Environment Food & Rural Affairs [DEFRA], 2024). The data are provided at 1km^2^ spatial resolution (as a csv file containing the x,y coordinates of each pixel centroid, and the modelled pollution values). We assigned codes to each NOx value, according to the LSOA it fell within. Where there were more than one NOx points within an LSOA, we produced a mean NOx value c.f. (Barnes et al., 2019). Where LSOAs had no NOx points directly within them, we used a form of nearest neighbour interpolation with a maximum distance of 499 metres to assign an appropriate NOx value. We removed all offshore values by specifying the processing extent to the terrestrial high tide limit.

**1b. Rescaling IMD 2019 and the Rural-Urban Classification to the 2021 LSOA census boundaries**

Analyses for this paper were performed at the 2021 LSOA scale. This is the scale used by the 2021 census and associated data. There are some changes in LSOA identifiers and boundaries between the 2011 and 2021 data, including both aggregation and disaggregation of LSOAs, which require attention when employing the 2019 IMD data which uses an older edition of the LSOA identifiers. We converted the IMD data from the 2011 to the 2021 LSOA geography using weighted averages of the IMD scores (overall and living environment domain). The weights are calculated using the population distributions within the intersections of the 2011 and 2011 LSOA polygons (Norman et al., 2024).

The rural-urban classification for England was produced using 2011 LSOA boundaries and has not been updated since. We adjusted these data to the 2021 LSOA boundaries so that they are comparable with the rest of the data. A similar process was followed as for the conversion of the IMD. Where there were intersections of differently categorised areas the rural-urban code of the largest area of overlap was allocated. This was the case for 79 (0.23%) of the 33,755 LSOAs. In the binary categories we use in the modelling, five locations change from being categorised as rural in the 2011 geography to being urban using the 2021 boundaries and seven locations change from being categorised as urban to being rural.

The rural-urban classification is provided in a 1-8 format, with four types of urban areas and four types of rural areas. As is common practice, we reclassified this measure into a binary measure: urban, rural.

1. **Sensitivity analyses**

In order to test the robustness of our analysis, we performed a series of sensitivity tests, the results of which are included here. First, we exclude London, due to its atypical relationship between access to public transport, emissions and area deprivation, and its unique demographics.

Second, we run the analysis using an alternative version of IMD. One of the seven domains included in the IMD relates to the living environment, which refers to both indoor and outdoor environments. The contribution of the living environment domain to the overall IMD score is one of the smallest of the seven domains which are each weighted as follows: income deprivation (22.5), employment deprivation (22.5), health deprivation and disability (13.5), education, skills and training deprivation (13.5), barriers to housing and services (9.3), crime (9.3), living environment deprivation (9.3). The living environment domain of the IMD is made up of four indicators; two reflecting indoor environments and two reflecting outdoor environments (emissions rates of nitrogen dioxide, benzene, sulphur dioxide and particulates, and traffic accidents involving pedestrians or cyclists). The final score for this domain is calculated by summing the sub-domains of outdoor and indoor living environment in a manner which allocates two thirds contribution to the indoor score, and one third to the outdoor score. Further detail on the composition and weighting of the other domains is available at (Ministry of Housing Communities and Local Government, 2019).

Though this domain receives one of the smallest weightings of the seven domains (making its contribution to the overall score relatively small) importantly, it does include a measure of ‘air quality’ alongside other indicators. Furthermore, though neither NOx nor sub-elements NO or NO2 are included in the outdoor pollution measure and this element of the domain has a relatively small contribution to its overall score, one might argue that NOx is likely to be correlated with the four pollutants which are included and also with the number of traffic accidents. As such, inequalities by IMD might be overstated given, in a sense, our outcome is included as part of the IMD variable. Previous ecological research on air pollution in England has re-calculated IMD without this domain included (Milojevic et al., 2017). Though Milojevic and co-authors find excluding this domain made little difference to their results, we also re-run our analysis with the living environment domain of the IMD excluded as a sensitivity analysis.

We recalculate the IMD score with this domain excluded following the process specified in Appendix B of the Research Report (Department for Communities and Local Government [DCLG], 2019). We then recalculate the 2019 IMD scores from 2011 to 2021 LSOA boundaries following the process outlined above.

Thirdly, we run the analysis using an alternate version of the IMD with the education domain excluded. Since education level is one of our stratum-defining variables, we wanted to ensure that by including IMD with this domain that we were not inadvertently ‘double counting’ education. We follow the same protocol for this sensitivity analysis as that for the IMD ‘living environment’ sensitivity analysis to remove this domain from the IMD variable.

Fourthly, we run a supplementary analysis with a population density measure (population per hectare) included. Whilst we do include a rural-urban indicator in our main analysis, we recognise that there is likely to be significant variation in population density within the broad category of ‘urban’. We run our primary model (2b) with population density included as a control variable.

Fifthly, and related to the previous analysis, we run a version of the model with only rural areas included. We do this to understand the role of urban centres in driving the results we find. It should be noted that this substantially changes the number of observations, the strata which can be produced and their number.

In our penultimate model, we re-run our primary models (2a and 2b) with a control for spatial autocorrelation. We do this using the coordinates of 2021 LSOA population weighted centroids (Office for National Statistics, 2023). We do this to investigate the underlying spatial relationships in the data.

Finally, we uncover patterns suggesting that places which have a high proportion of the population who are minority ethnic, not ageing, urban and with medium to high proportions of the population having a degree are places with the highest average concentrations of NOx. Having examined the location of these places, we hypothesised that these may be university towns. We downloaded LSOA level 2021 census data from on economic activity Nomis (Nomis, 2025) and examined the proportion of the population aged over 16 who are full time students. We compared the mean and distribution of the full time student population across all LSOAs, and across LSOAs in our three strata with the highest average NOx scores: 33210, 23310, 33310.

1. **Sensitivity analyses results**

Firstly, we excluded London from our analysis due to its atypical relationship with the other variables in the model compared to the rest of England. In this analysis, we find the strata with the highest estimated NOx concentration remains the same (33310) as in Model 2b in the main analysis (results shown in Table A5). Though the exact order of the strata varies, the top 10 strata remain entirely high proportion minority ethnic, and almost entirely urban, with the bottom 10 remaining entirely low proportion minority ethnic and rural. This suggests London-specific trends are not driving overall patterns of estimated NOx. However, examining the statistically significant residuals from the second (additive) model without London reveals a much-reduced residual for strata 33310 (most deprived, high education, urban and not ageing) though still highest. Notably, the sample size for this stratum is greatly reduced in the model excluding London (139 versus 498). This might suggest LSOAs belonging to this stratum are more likely to be found in London and those have higher NOx concentrations compared to LSOAs elsewhere.

Secondly, we re-ran the analysis with an alternative version of the IMD 2019 measure with the living environment domain excluded. This analysis produced very similar results, suggesting the inclusion of the living environment domain is not driving the results we find (results in Table A6). Similarly, our sensitivity analysis excluding the education domain of the IMD also produced similar results to the main analyses (2a and 2b) in the main manuscript (results in Table A7). Notably, the education variable remains not statistically significant.

We then ran a supplementary analysis with population density included. We find that population density is associated with NOx, such that more densely populated places are more likely to have higher NOx concentrations (0.077*** CI [0.075 0.079]). Including this variable in the model explains more of the variance of the model (PCV of 89.76% vs 79.26% in the comparable main model of the manuscript) but a notable portion remains (VPC = 8.11%). Furthermore, the coefficient direction and strength for each of the stratum-defining variables in the model are changed minimally by the inclusion of this variable. This would suggest that population density does not explain the inequalities we observe by ethnicity, urbanity and age. Relatedly, we also run a supplementary analysis with all urban areas removed in order to determine the role of urban centres in driving the results. We find the age variable is no longer statistically significant, but that the ethnicity effect remains similar in size, direction and statistical significance as the main analysis (2b) in the manuscript. This again suggests that inequalities we observe are not entirely being driven by the role of urban centres.

In our penultimate sensitivity analysis, we control for spatial autocorrelation by using co-ordinates for population weighted centroids of LSOAs and including an exponential spatial correlation structure (using nlme in R). In this analysis, estimates remain similar in direction and size for the ethnicity, age and urbanity variables. This suggest that our main results are robust to potential spatial autocorrelation. However, in addition in this model we find that education becomes statistically significant, with places with higher average education having average lower NOx concentrations. Furthermore, the middle category only of IMD also becomes statistically significant, though with a small effect size. Interestingly, the stratum-level variance reduced to zero in both models. The modelling of spatial autocorrelation ‘controls out’ all stratum inequalities not present in the main effects, because those strata are themselves spatially situated. While useful as a sensitivity analysis for understanding the role of underlying spatial relationships in our findings, ultimately, accounting for spatial autocorrelation in our residuals controls out the very thing that we are interested in. However, we do acknowledge that spatial autocorrelation is an important potential issue in analyses using EIM. Further research could perhaps look at the spatial patterning of the inequalities we find and any potential spillover effects of this.

Finally, we examined the proportions of students in fulltime education across LSOAs across England in the three strata with the highest average NOx estimates. We find minimal differences between these three strata and the rest. For all LSOAs in England, the mean percentage of full-time students is 7.42, SD 7.13, range 1.05-90.36. For the three strata with the highest NOx values, the LSOA average percentage of full-time students is 11.40, SD 8.46, range 1.69-83.15. Whilst there is a small difference between the means, the standard deviation and range remain similar, meaning the areas captured by the three strata are not areas particularly notable for a very high proportion of students. This suggests that university towns do not explain the higher NOx concentrations in these strata. Furthermore, it also suggests that places with these stratum characteristics (which have a high proportion of the population who are minority ethnic, not ageing, urban and with medium to high proportions of the population having a degree) do not necessarily have transient student populations.

**Supplementary tables and figures**

**Table A1 – Strata containing zero LSOAs**

| **Stratum id** | **IMD** | **Ethnicity** | **Education** | **Rural-Urban** | **Age** |
| --- | --- | --- | --- | --- | --- |
| 13101 | Least deprived | High % minority ethnic | Low | Rural | Ageing |
| 23101 | Middle deprived | High % minority ethnic | Low | Rural | Ageing |
| 31300 | Most deprived | Low % minority ethnic | High | Rural | Not ageing |
| 31301 | Most deprived | Low % minority ethnic | High | Rural | Ageing |
| 31311 | Most deprived | Low % minority ethnic | High | Urban | Ageing |
| 32300 | Most deprived | Mid % minority ethnic | High | Rural | Not ageing |
| 32301 | Most deprived | Mid % minority ethnic | High | Rural | Ageing |
| 33100 | Most deprived | High % minority ethnic | Low | Rural | Not ageing |
| 33101 | Most deprived | High % minority ethnic | Low | Rural | Ageing |
| 33200 | Most deprived | High % minority ethnic | Mid | Rural | Not ageing |
| 33201 | Most deprived | High % minority ethnic | Mid | Rural | Ageing |
| 33300 | Most deprived | High % minority ethnic | High | Rural | Not ageing |
| 33301 | Most deprived | High % minority ethnic | High | Rural | Ageing |
| 33311 | Most deprived | High % minority ethnic | High | Urban | Ageing |

**Table A2** – Highest vs lowest 10 ranked predicted stratum NOx concentration means (Model 2b)

| **Stratum number** | **IMD** | **Tertiles of % population minority ethnic** | **Tertiles of % population L4 educated** | **Rural- Urban** | **Binary indicator of 25% of the population aged 65+** | **Stratum sample size** | **Rank** | **Predicted means** | **95%CI** |  |
| --- | --- | --- | --- | --- | --- | --- | --- | --- | --- | --- |
| **Top 10 strata** | | |  |  |  |  |  |  |  |  |
| 33310 | most dep | hi eth | hi educ | Urb | Not ageing | 498 | 1 | 42.91 | 40.75 | 45.07 |
| 23310 | mid dep | hi eth | hi educ | Urb | Not ageing | 3624 | 2 | 36.82 | 35.06 | 38.59 |
| 33210 | most dep | hi eth | med educ | Urb | Not ageing | 900 | 3 | 33.04 | 31.04 | 35.04 |
| 23210 | mid dep | hi eth | med educ | Urb | Not ageing | 2146 | 4 | 28.69 | 26.90 | 30.49 |
| 13310 | least dep | hi eth | hi educ | Urb | Not ageing | 853 | 5 | 27.29 | 25.46 | 29.12 |
| 33110 | most dep | hi eth | low educ | Urb | Not ageing | 1907 | 6 | 26.65 | 24.71 | 28.58 |
| 33111 | most dep | hi eth | low educ | Urb | Ageing | 5 | 7 | 25.86 | 21.32 | 30.41 |
| 33211 | most dep | hi eth | med educ | Urb | Ageing | 4 | 8 | 25.81 | 21.09 | 30.54 |
| 23110 | mid dep | hi eth | low educ | Urb | Not ageing | 738 | 9 | 25.40 | 23.50 | 27.30 |
| 23200 | mid dep | hi eth | med educ | Rur | Not ageing | 15 | 10 | 25.30 | 21.77 | 28.83 |
| **Bottom 10 strata** | | |  |  |  |  |  |  |  |  |
| 21100 | mid dep | low eth | low educ | Rur | Not ageing | 364 | 85 | 11.46 | 9.58 | 13.34 |
| 21300 | mid dep | low eth | hi educ | Rur | Not ageing | 295 | 86 | 10.86 | 8.91 | 12.81 |
| 21200 | mid dep | low eth | med educ | Rur | Not ageing | 536 | 87 | 10.85 | 9.01 | 12.68 |
| 31100 | most dep | low eth | low educ | Rur | Not ageing | 123 | 88 | 10.79 | 8.51 | 13.06 |
| 21101 | mid dep | low eth | low educ | Rur | Ageing | 423 | 89 | 10.74 | 8.87 | 12.61 |
| 31201 | most dep | low eth | med educ | Rur | Ageing | 6 | 90 | 10.71 | 6.31 | 15.11 |
| 11301 | least dep | low eth | hi educ | Rur | Ageing | 389 | 91 | 10.49 | 8.56 | 12.42 |
| 31101 | most dep | low eth | low educ | Rur | Ageing | 36 | 92 | 10.22 | 7.30 | 13.14 |
| 21201 | mid dep | low eth | med educ | Rur | Ageing | 1042 | 93 | 9.39 | 7.60 | 11.18 |
| 21301 | mid dep | low eth | hi educ | Rur | Ageing | 660 | 94 | 8.63 | 6.75 | 10.51 |

**Table A3 - Table 4: Rank of statistically significant residuals from Model 2b**

| **Stratum number** | **IMD** | **Tertiles of % population minority ethnic** | **Tertiles of % population L4 educated** | **Rural- Urban** | **Binary indicator of 25% of the population aged 65+** | **Rank of residual** | **Residual** | **95% CI** |  |
| --- | --- | --- | --- | --- | --- | --- | --- | --- | --- |
| 33310 | most dep | hi eth | hi educ | Urb | Not ageing | 1 | 14.39 | 15.00 | 13.78 |
| 23310 | mid dep | hi eth | hi educ | Urb | Not ageing | 2 | 9.46 | 9.69 | 9.23 |
| 33210 | most dep | hi eth | med educ | Urb | Not ageing | 3 | 4.90 | 5.35 | 4.44 |
| 11101 | least dep | low eth | low educ | Rur | Ageing | 4 | 3.70 | 6.31 | 1.08 |
| 23200 | mid dep | hi eth | med educ | Rur | Not ageing | 5 | 3.38 | 6.34 | 0.43 |
| 12111 | least dep | med eth | low educ | Urb | Ageing | 6 | 2.89 | 4.86 | 0.91 |
| 22101 | mid dep | med eth | low educ | Rur | Ageing | 7 | 2.87 | 5.36 | 0.39 |
| 11201 | least dep | low eth | med educ | Rur | Ageing | 8 | 2.69 | 3.65 | 1.74 |
| 12201 | least dep | med eth | med educ | Rur | Ageing | 9 | 2.63 | 5.11 | 0.14 |
| 23210 | mid dep | hi eth | med educ | Urb | Not ageing | 10 | 1.71 | 2.01 | 1.41 |
| 11200 | least dep | low eth | med educ | Rur | Not ageing | 11 | 1.44 | 2.55 | 0.33 |
| 11111 | least dep | low eth | low educ | Urb | Ageing | 12 | 1.43 | 2.70 | 0.16 |
| 22111 | mid dep | med eth | low educ | Urb | Ageing | 13 | 1.36 | 2.13 | 0.59 |
| 21101 | mid dep | low eth | low educ | Rur | Ageing | 14 | 1.33 | 2.00 | 0.67 |
| 11301 | least dep | low eth | hi educ | Rur | Ageing | 15 | 1.20 | 1.89 | 0.51 |
| 11300 | least dep | low eth | hi educ | Rur | Not ageing | 16 | 1.13 | 2.14 | 0.11 |
| 21111 | mid dep | low eth | low educ | Urb | Ageing | 17 | 1.07 | 1.53 | 0.62 |
| 11211 | least dep | low eth | med educ | Urb | Ageing | 18 | 0.80 | 1.44 | 0.16 |
| 22210 | mid dep | med eth | med educ | Urb | Not ageing | 19 | -0.41 | -0.08 | -0.73 |
| 12210 | least dep | med eth | med educ | Urb | Not ageing | 20 | -0.67 | -0.10 | -1.25 |
| 11210 | least dep | low eth | med educ | Urb | Not ageing | 21 | -0.87 | -0.05 | -1.70 |
| 21210 | mid dep | low eth | med educ | Urb | Not ageing | 22 | -0.98 | -0.51 | -1.44 |
|  |  |  |  |  |  |  |  |  |  |
| 11310 | least dep | low eth | hi educ | Urb | Not ageing | 23 | -1.15 | -0.24 | -2.06 |
| 21301 | mid dep | low eth | hi educ | Rur | Ageing | 24 | -1.15 | -0.62 | -1.68 |
| 12311 | least dep | med eth | hi educ | Urb | Ageing | 25 | -1.25 | -0.65 | -1.85 |
| 33110 | most dep | hi eth | low educ | Urb | Not ageing | 26 | -1.50 | -1.19 | -1.82 |
| 22300 | mid dep | med eth | hi educ | Rur | Not ageing | 27 | -1.59 | -0.64 | -2.53 |
| 23110 | mid dep | hi eth | low educ | Urb | Not ageing | 28 | -1.59 | -1.09 | -2.10 |
| 31100 | most dep | low eth | low educ | Rur | Not ageing | 29 | -1.62 | -0.41 | -2.82 |
| 13311 | least dep | hi eth | hi educ | Urb | Ageing | 30 | -1.85 | -0.73 | -2.97 |
| 23211 | mid dep | hi eth | med educ | Urb | Ageing | 31 | -1.96 | -0.32 | -3.59 |
| 21311 | mid dep | low eth | hi educ | Urb | Ageing | 32 | -1.96 | -0.85 | -3.07 |
| 21310 | mid dep | low eth | hi educ | Urb | Not ageing | 33 | -1.97 | -0.86 | -3.08 |
| 13210 | least dep | hi eth | med educ | Urb | Not ageing | 34 | -1.99 | -0.82 | -3.17 |
| 23311 | mid dep | hi eth | hi educ | Urb | Ageing | 35 | -2.03 | -0.46 | -3.60 |
| 32211 | most dep | med eth | med educ | Urb | Ageing | 36 | -3.25 | -0.48 | -6.02 |
| 23300 | mid dep | hi eth | hi educ | Rur | Not ageing | 37 | -3.31 | -1.20 | -5.41 |
| 13110 | least dep | hi eth | low educ | Urb | Not ageing | 38 | -3.63 | -0.36 | -6.90 |
| 13300 | least dep | hi eth | hi educ | Rur | Not ageing | 39 | -4.10 | -1.73 | -6.47 |
| 13100 | least dep | hi eth | low educ | Rur | Not ageing | 40 | -4.29 | -0.42 | -8.15 |

**Table: A4 – All predicted values and their rank (Model 2b)**

| stratum | IMD | Tertiles of % population minority ethnic | Tertiles of % population L4 educated | Rural/ Urban | Binary indicator of 25% of the population aged 65+ | n | rank | Predicted Values (M2) | 95%CI |  |
| --- | --- | --- | --- | --- | --- | --- | --- | --- | --- | --- |
| 21301 | 2 | 1 | 3 | 0 | 1 | 660 | 1 | 8.634 | 6.754 | 10.513 |
| 21201 | 2 | 1 | 2 | 0 | 1 | 1042 | 2 | 9.391 | 7.602 | 11.181 |
| 31101 | 3 | 1 | 1 | 0 | 1 | 36 | 3 | 10.218 | 7.295 | 13.140 |
| 11301 | 1 | 1 | 3 | 0 | 1 | 389 | 4 | 10.491 | 8.558 | 12.424 |
| 31201 | 3 | 1 | 2 | 0 | 1 | 6 | 5 | 10.712 | 6.309 | 15.114 |
| 21101 | 2 | 1 | 1 | 0 | 1 | 423 | 6 | 10.743 | 8.875 | 12.610 |
| 31100 | 3 | 1 | 1 | 0 | 0 | 123 | 7 | 10.790 | 8.515 | 13.064 |
| 21200 | 2 | 1 | 2 | 0 | 0 | 536 | 8 | 10.847 | 9.010 | 12.683 |
| 21300 | 2 | 1 | 3 | 0 | 0 | 295 | 9 | 10.861 | 8.912 | 12.810 |
| 21100 | 2 | 1 | 1 | 0 | 0 | 364 | 10 | 11.458 | 9.579 | 13.337 |
| 11201 | 1 | 1 | 2 | 0 | 1 | 200 | 11 | 11.602 | 9.591 | 13.614 |
| 31200 | 3 | 1 | 2 | 0 | 0 | 6 | 12 | 12.024 | 7.663 | 16.385 |
| 11200 | 1 | 1 | 2 | 0 | 0 | 146 | 13 | 12.187 | 10.104 | 14.270 |
| 11300 | 1 | 1 | 3 | 0 | 0 | 177 | 14 | 12.256 | 10.212 | 14.301 |
| 11101 | 1 | 1 | 1 | 0 | 1 | 21 | 15 | 12.613 | 9.416 | 15.811 |
| 22301 | 2 | 2 | 3 | 0 | 1 | 76 | 16 | 12.623 | 10.249 | 14.997 |
| 32101 | 3 | 2 | 1 | 0 | 1 | 3 | 17 | 12.714 | 7.812 | 17.615 |
| 11100 | 1 | 1 | 1 | 0 | 0 | 9 | 18 | 12.877 | 8.952 | 16.802 |
| 21311 | 2 | 1 | 3 | 1 | 1 | 146 | 19 | 12.892 | 10.773 | 15.011 |
| 22201 | 2 | 2 | 2 | 0 | 1 | 81 | 20 | 13.203 | 10.884 | 15.522 |
| 32201 | 3 | 2 | 2 | 0 | 1 | 1 | 21 | 13.552 | 8.135 | 18.969 |
| 12301 | 1 | 2 | 3 | 0 | 1 | 112 | 22 | 13.670 | 11.428 | 15.912 |
| 22300 | 2 | 2 | 3 | 0 | 0 | 205 | 23 | 13.703 | 11.692 | 15.714 |
| 11311 | 1 | 1 | 3 | 1 | 1 | 410 | 24 | 13.753 | 11.851 | 15.654 |
| 31211 | 3 | 1 | 2 | 1 | 1 | 9 | 25 | 14.039 | 10.051 | 18.028 |
| 21211 | 2 | 1 | 2 | 1 | 1 | 724 | 26 | 14.046 | 12.230 | 15.862 |
| 12100 | 1 | 2 | 1 | 0 | 0 | 4 | 27 | 14.456 | 9.835 | 19.078 |
| 12300 | 1 | 2 | 3 | 0 | 0 | 219 | 28 | 14.506 | 12.497 | 16.516 |
| 21310 | 2 | 1 | 3 | 1 | 0 | 147 | 29 | 14.723 | 12.576 | 16.870 |
| 11211 | 1 | 1 | 2 | 1 | 1 | 459 | 30 | 14.774 | 12.916 | 16.631 |
| 31111 | 3 | 1 | 1 | 1 | 1 | 107 | 31 | 14.784 | 12.521 | 17.046 |
| 12101 | 1 | 2 | 1 | 0 | 1 | 2 | 32 | 14.796 | 9.732 | 19.860 |
| 11210 | 1 | 1 | 2 | 1 | 0 | 273 | 33 | 14.939 | 12.966 | 16.913 |
| 22200 | 2 | 2 | 2 | 0 | 0 | 250 | 34 | 15.030 | 13.072 | 16.989 |
| 11310 | 1 | 1 | 3 | 1 | 0 | 220 | 35 | 15.049 | 13.027 | 17.072 |
| 32100 | 3 | 2 | 1 | 0 | 0 | 17 | 36 | 15.081 | 11.628 | 18.534 |
| 12200 | 1 | 2 | 2 | 0 | 0 | 87 | 37 | 15.125 | 12.831 | 17.420 |
| 12201 | 1 | 2 | 2 | 0 | 1 | 24 | 38 | 15.201 | 12.108 | 18.295 |
| 21210 | 2 | 1 | 2 | 1 | 0 | 857 | 39 | 15.331 | 13.469 | 17.192 |
| 11111 | 1 | 1 | 1 | 1 | 1 | 111 | 40 | 15.418 | 13.212 | 17.623 |
| 22100 | 2 | 2 | 1 | 0 | 0 | 108 | 41 | 15.530 | 13.320 | 17.739 |
| 21111 | 2 | 1 | 1 | 1 | 1 | 908 | 42 | 15.551 | 13.755 | 17.347 |
| 31210 | 3 | 1 | 2 | 1 | 0 | 24 | 43 | 15.939 | 12.802 | 19.076 |
| 22101 | 2 | 2 | 1 | 0 | 1 | 24 | 44 | 15.949 | 12.858 | 19.040 |
| 32200 | 3 | 2 | 2 | 0 | 0 | 5 | 45 | 15.953 | 11.439 | 20.467 |
| 32211 | 3 | 2 | 2 | 1 | 1 | 18 | 46 | 16.046 | 12.659 | 19.433 |
| 21110 | 2 | 1 | 1 | 1 | 0 | 1324 | 47 | 16.161 | 14.334 | 17.987 |
| 31310 | 3 | 1 | 3 | 1 | 0 | 2 | 48 | 16.645 | 11.522 | 21.767 |
| 11110 | 1 | 1 | 1 | 1 | 0 | 51 | 49 | 16.777 | 14.193 | 19.361 |
| 12311 | 1 | 2 | 3 | 1 | 1 | 518 | 50 | 16.780 | 14.917 | 18.643 |
| 13100 | 1 | 3 | 1 | 0 | 0 | 6 | 51 | 17.144 | 12.736 | 21.552 |
| 31110 | 3 | 1 | 1 | 1 | 0 | 1047 | 52 | 17.153 | 15.297 | 19.009 |
| 13300 | 1 | 3 | 3 | 0 | 0 | 27 | 53 | 17.701 | 14.626 | 20.776 |
| 22211 | 2 | 2 | 2 | 1 | 1 | 389 | 54 | 17.822 | 15.955 | 19.689 |
| 22311 | 2 | 2 | 3 | 1 | 1 | 196 | 55 | 17.855 | 15.840 | 19.870 |
| 12211 | 1 | 2 | 2 | 1 | 1 | 237 | 56 | 18.267 | 16.302 | 20.233 |
| 32311 | 3 | 2 | 3 | 1 | 1 | 2 | 57 | 18.361 | 13.227 | 23.494 |
| 32111 | 3 | 2 | 1 | 1 | 1 | 47 | 58 | 18.608 | 15.946 | 21.271 |
| 12210 | 1 | 2 | 2 | 1 | 0 | 566 | 59 | 18.808 | 16.947 | 20.670 |
| 13200 | 1 | 3 | 2 | 0 | 0 | 7 | 60 | 18.910 | 14.686 | 23.134 |
| 23100 | 2 | 3 | 1 | 0 | 0 | 10 | 61 | 18.977 | 15.059 | 22.895 |
| 23300 | 2 | 3 | 3 | 0 | 0 | 36 | 62 | 18.990 | 16.142 | 21.838 |
| 13301 | 1 | 3 | 3 | 0 | 1 | 1 | 63 | 19.065 | 13.657 | 24.473 |
| 22111 | 2 | 2 | 1 | 1 | 1 | 312 | 64 | 19.508 | 17.578 | 21.438 |
| 12310 | 1 | 2 | 3 | 1 | 0 | 1220 | 65 | 19.518 | 17.719 | 21.316 |
| 22210 | 2 | 2 | 2 | 1 | 0 | 1826 | 66 | 19.566 | 17.779 | 21.353 |
| 12110 | 1 | 2 | 1 | 1 | 0 | 56 | 67 | 19.683 | 17.138 | 22.227 |
| 13201 | 1 | 3 | 2 | 0 | 1 | 1 | 68 | 19.719 | 14.303 | 25.135 |
| 23301 | 2 | 3 | 3 | 0 | 1 | 1 | 69 | 19.965 | 14.575 | 25.355 |
| 22110 | 2 | 2 | 1 | 1 | 0 | 1638 | 70 | 20.036 | 18.221 | 21.852 |
| 12111 | 1 | 2 | 1 | 1 | 1 | 42 | 71 | 20.538 | 17.831 | 23.245 |
| 32210 | 3 | 2 | 2 | 1 | 0 | 199 | 72 | 20.600 | 18.494 | 22.707 |
| 22310 | 2 | 2 | 3 | 1 | 0 | 984 | 73 | 20.711 | 18.888 | 22.534 |
| 32110 | 3 | 2 | 1 | 1 | 0 | 1764 | 74 | 20.934 | 19.102 | 22.767 |
| 32310 | 3 | 2 | 3 | 1 | 0 | 20 | 75 | 21.619 | 18.267 | 24.971 |
| 23201 | 2 | 3 | 2 | 0 | 1 | 2 | 76 | 21.716 | 16.599 | 26.833 |
| 13211 | 1 | 3 | 2 | 1 | 1 | 17 | 77 | 22.614 | 19.192 | 26.037 |
| 13110 | 1 | 3 | 1 | 1 | 0 | 11 | 78 | 22.868 | 19.067 | 26.670 |
| 13311 | 1 | 3 | 3 | 1 | 1 | 143 | 79 | 23.186 | 20.973 | 25.398 |
| 23211 | 2 | 3 | 2 | 1 | 1 | 64 | 80 | 23.188 | 20.696 | 25.680 |
| 23311 | 2 | 3 | 3 | 1 | 1 | 70 | 81 | 23.496 | 21.046 | 25.947 |
| 13210 | 1 | 3 | 2 | 1 | 0 | 131 | 82 | 24.496 | 22.335 | 26.657 |
| 23111 | 2 | 3 | 1 | 1 | 1 | 33 | 83 | 24.727 | 21.804 | 27.651 |
| 13111 | 1 | 3 | 1 | 1 | 1 | 1 | 84 | 25.140 | 19.764 | 30.516 |
| 23200 | 2 | 3 | 2 | 0 | 0 | 15 | 85 | 25.297 | 21.766 | 28.829 |
| 23110 | 2 | 3 | 1 | 1 | 0 | 738 | 86 | 25.400 | 23.500 | 27.299 |
| 33211 | 3 | 3 | 2 | 1 | 1 | 4 | 87 | 25.814 | 21.092 | 30.535 |
| 33111 | 3 | 3 | 1 | 1 | 1 | 5 | 88 | 25.862 | 21.317 | 30.407 |
| 33110 | 3 | 3 | 1 | 1 | 0 | 1907 | 89 | 26.648 | 24.713 | 28.583 |
| 13310 | 1 | 3 | 3 | 1 | 0 | 853 | 90 | 27.293 | 25.464 | 29.122 |
| 23210 | 2 | 3 | 2 | 1 | 0 | 2146 | 91 | 28.692 | 26.895 | 30.488 |
| 33210 | 3 | 3 | 2 | 1 | 0 | 900 | 92 | 33.037 | 31.038 | 35.037 |
| 23310 | 2 | 3 | 3 | 1 | 0 | 3624 | 93 | 36.824 | 35.058 | 38.590 |
| 33310 | 3 | 3 | 3 | 1 | 0 | 498 | 94 | 42.911 | 40.749 | 45.074 |
|  | | | | | | | | | | |

Figure A1

Sensitivity analyses results

| **Table A5 – Model 2a and 2b comparison – London excluded** | | |
| --- | --- | --- |
|  | **2a** | **2b** |
| **Alternative IMD 2019**  **(Ref: least deprived)** |  |  |
| **Mid deprived** |  | 0.221 |
|  |  | [-0.478 0.919] |
| **Most deprived** |  | 0.866 |
|  |  | [-0.038 1.770] |
| **Tertiles of % population minority ethnic**  **(Ref: low % minority ethnic)** |  |  |
| **Medium % minority ethnic** |  | 3.685*** |
|  |  | [2.979 4.391] |
| **High % minority ethnic** |  | 8.548*** |
|  |  | [7.701 9.394] |
| **Tertiles of % population L4 educated**  **(Ref: low education)** |  |  |
| **Medium education** |  | -0.481 |
|  |  | [-1.244 0.282] |
| **High education** |  | -1.047* |
|  |  | [-1.848 -0.246] |
| **Rural-Urban classification**  **(Ref: rural)** |  |  |
| **Urban** |  | 4.293*** |
|  |  | [3.635 4.952] |
| **Binary indicator of 25% of the population aged 65+**  **(Ref: not ageing)** |  |  |
| **Ageing** |  | -1.313*** |
|  |  | [-1.949 -0.677] |
| **Intercept** | 16.906*** | 11.737*** |
|  | [15.930 17.881] | [10.802 12.672] |
| **Between-stratum variance** | 21.320 | 1.649 |
| **Within-stratum variance** | 24.980 | 25.003 |
| **Number of observations** | 28761 | 28761 |
| **AIC** | 174574.4 | 174418.6 |
| **VPC** | 46.05% | 6.19% |
| **PCV** |  | 92.27% |
| ***p<0.05, **p<0.01, ***p<0.001** |  |  |

| **Table A6 – Models 2a and 2b comparison – alternative version of IMD (without living environment domain)** | | |
| --- | --- | --- |
|  | **2a** | **2b** |
| **Alternative IMD 2019**  **(Ref: least deprived)** |  |  |
| **Mid deprived** |  | 0.336 |
|  |  | [-1.063 1.734] |
| **Most deprived** |  | 1.547 |
|  |  | [-0.203 3.296] |
| **Tertiles of % population minority ethnic**  **(Ref: low % minority ethnic)** |  |  |
| **Medium % minority ethnic** |  | 3.717*** |
|  |  | [2.293 5.142] |
| **High % minority ethnic** |  | 10.824*** |
|  |  | [9.183 12.464] |
| **Tertiles of % population L4 educated**  **(Ref: low education)** |  |  |
| **Medium education** |  | 0.122 |
|  |  | [-1.380 1.624] |
| **High education** |  | 0.579 |
|  |  | [-1.014 2.172] |
| **Rural-Urban classification**  **(Ref: rural)** |  |  |
| **Urban** |  | 5.172*** |
|  |  | [3.875 6.469] |
| **Binary indicator of 25% of the population aged 65+**  **(Ref: not ageing)** |  |  |
| **Ageing** |  | -1.916** |
|  |  | [-3.173 -0.658] |
| **Intercept** | 17.656*** | 10.691*** |
|  | [16.366 18.946] | [8.853 12.530] |
| **Between-stratum variance** | 36.806 | 7.266 |
| **Within-stratum variance** | 50.713 | 50.720 |
| **Number of observations** | 33755 | 33755 |
| **AIC** | 228710.6 | 228601.4 |
| **VPC** | 42.05% | 12.53% |
| **PCV** |  | 80.26% |
| ***p<0.05, **p<0.01, ***p<0.001** |  |  |

**Table A7 - Models 2a and 2b comparison – alternative version of IMD (without education domain)**

|  | | |
| --- | --- | --- |
|  | **2a** | **2b** |
| **Alternative IMD 2019**  **(Ref: least deprived)** |  |  |
| **Mid deprived** |  | 0.494 |
|  |  | [-0.902 1.891] |
| **Most deprived** |  | 1.818* |
|  |  | [0.091 3.545] |
| **Tertiles of % population minority ethnic**  **(Ref: low % minority ethnic)** |  |  |
| **Medium % minority ethnic** |  | 3.703*** |
|  |  | [2.275 5.131] |
| **High % minority ethnic** |  | 10.629*** |
|  |  | [8.980 12.278] |
| **Tertiles of % population L4 educated**  **(Ref: low education)** |  |  |
| **Medium education** |  | -0.046 |
|  |  | [-1.538 1.445] |
| **High education** |  | 0.374 |
|  |  | [-1.204 1.952] |
| **Rural-Urban classification**  **(Ref: rural)** |  |  |
| **Urban** |  | 5.056*** |
|  |  | [3.755 6.357] |
| **Binary indicator of 25% of the population aged 65+**  **(Ref: not ageing)** |  |  |
| **Ageing** |  | -1.831** |
|  |  | [-3.091 -0.571] |
| **Intercept** | 17.584*** | 10.728*** |
|  | [16.313 18.856] | [8.941 12.515] |
| **Between-stratum variance** | 36.366 | 7.591 |
| **Within-stratum variance** | 48.414 | 48.416 |
| **Number of observations** | 33755 | 33755 |
| **AIC** | 227157 | 227046.7 |
| **VPC** | 42.89% | 13.55% |
| **PCV** |  | 79.12% |
| ***p<0.05, **p<0.01, ***p<0.001** |  |  |

**Table A8 - Models 2a and 2b comparison – including population density as a control variable**

|  | | |
| --- | --- | --- |
|  | **2a** | **2b** |
| **IMD 2019**  **(Ref: least deprived)** |  |  |
| **Mid deprived** |  | 0.186 |
|  |  | [-0.828 1.200] |
| **Most deprived** |  | 0.401 |
|  |  | [-0.892 1.693] |
| **Tertiles of % population minority ethnic**  **(Ref: low % minority ethnic)** |  |  |
| **Medium % minority ethnic** |  | 3.195*** |
|  |  | [2.164 4.226] |
| **High % minority ethnic** |  | 9.319*** |
|  |  | [8.107 10.531] |
| **Tertiles of % population L4 educated**  **(Ref: low education)** |  |  |
| **Medium education** |  | 0.027 |
|  |  | [-1.074 1.128] |
| **High education** |  | 0.357 |
|  |  | [-0.800 1.514] |
| **Rural-Urban classification**  **(Ref: rural)** |  |  |
| **Urban** |  | 2.949*** |
|  |  | [1.996 3.902] |
| **Binary indicator of 25% of the population aged 65+**  **(Ref: not ageing)** |  |  |
| **Ageing** |  | -1.027* |
|  |  | [-1.947 -0.107] |
| **People per hectare** |  | 0.077*** |
|  |  | [0.075 0.079] |
| **Intercept** | 17.576*** | 10.376*** |
|  | [16.312 18.839] | [9.030 11.721] |
| **Between-stratum variance** | 35.659 | 3.652 |
| **Within-stratum variance** | 49.203 | 41.397 |
| **Number of observations** | 33755 | 33755 |
| **AIC** | 227693.2 | 221714.8 |
| **VPC** | 42.02% | 8.11% |
| **PCV** |  | 89.76% |
| ***p<0.05, **p<0.01, ***p<0.001** |  |  |

**Table A9 - Models 2a and 2b comparison – Analysing rural areas only**

|  | | |
| --- | --- | --- |
|  | **2a** | **2b** |
| **IMD 2019**  **(Ref: least deprived)** |  |  |
| **Mid deprived** |  | -0.134 |
|  |  | [-1.513 1.246] |
| **Most deprived** |  | -1.619 |
|  |  | [-3.630 0.392] |
| **Tertiles of % population minority ethnic**  **(Ref: low % minority ethnic)** |  |  |
| **Medium % minority ethnic** |  | 3.343*** |
|  |  | [1.952 4.735] |
| **High % minority ethnic** |  | 7.129??? |
|  |  | [5.240 9.017] |
| **Tertiles of % population L4 educated**  **(Ref: low education)** |  |  |
| **Medium education** |  | 0.445 |
|  |  | [-1.092 1.982] |
| **High education** |  | -1.498 |
|  |  | [-3.168 0.172] |
| **Binary indicator of 25% of the population aged 65+**  **(Ref: not ageing)** |  |  |
| **Ageing** |  | -0.554 |
|  |  | [-1.866 0.757] |
| **Intercept** | 14.017*** | 12.110*** |
|  | [12.862 15.172] | [10.374 13.846] |
| **Between-stratum variance** | 13.053 | 3.331 |
| **Within-stratum variance** | 12.861 | 12.877 |
| **Number of observations** | 5757 | 5757 |
| **AIC** | 229221.2 | 229162.6 |
| **VPC** | 44.87% | 15.35% |
| **PCV** |  | 77.72% |
| ***p<0.05, **p<0.01, ***p<0.001** |  |  |

**Table A10 - Models 2a and 2b comparison – modelled with a spatially autocorrelated error structure**

|  | | |
| --- | --- | --- |
|  | **2a** | **2b** |
| **IMD 2019**  **(Ref: least deprived)** |  |  |
| **Mid deprived** |  | -0.64*** |
|  |  | [-0.92   -0.35] |
| **Most deprived** |  | 0.36 |
|  |  | [-0.10   0.81] |
| **Tertiles of % population minority ethnic**  **(Ref: low % minority ethnic)** |  |  |
| **Medium % minority ethnic** |  | 3.33*** |
|  |  | [3.05  3.61] |
| **High % minority ethnic** |  | 8.20*** |
|  |  | [7.78   8.61] |
| **Tertiles of % population L4 educated**  **(Ref: low education)** |  |  |
| **Medium education** |  | -0.33* |
|  |  | [-0.64   -0.01] |
| **High education** |  | -0.57*** |
|  |  | [-0.92   -0.23] |
| **Rural-Urban classification**  **(Ref: rural)** |  |  |
| **Urban** |  | 3.32*** |
|  |  | [3.04   3.60] |
| **Binary indicator of 25% of the population aged 65+**  **(Ref: not ageing)** |  |  |
| **Ageing** |  | -0.49*** |
|  |  |  |
| **Intercept** | 14.78*** | 11.51*** |
|  | [14.60   14.95] | [11.09   11.93] |
| **Between-stratum variance** | 0.00 | 0.00 |
| **Within-stratum variance** | 41.59 | 27.44 |
| **Number of observations** | 33755 | 33755 |
| **AIC** |  |  |
| **VPC** | 0.00% | 0.00% |
| **PCV** |  | n/a |
| ***p<0.05, **p<0.01, ***p<0.001** |  |  |

**References**

Barnes, J. H., Chatterton, T. J. & Longhurst, J. W. 2019. Emissions vs exposure: Increasing injustice from road traffic-related air pollution in the United Kingdom. *Transportation research part D: transport and environment,* 73**,** 56-66.

Department for Communities and Local Government [Dclg]. 2019. *The English Indices of Deprivation 2019: Research report* [Online]. Available: <https://www.gov.uk/government/publications/english-indices-of-deprivation-2019-research-report#:~:text=The%20English%20indices%20of%20deprivation,the%20indices%20of%20deprivation%202019>. [Accessed 14/03/2024].

Department for Environment Food & Rural Affairs [Defra]. 2024. *Modelled background pollution data* [Online]. Available: <https://uk-air.defra.gov.uk/data/pcm-data> [Accessed 15/03/2024].

Milojevic, A., Niedzwiedz, C. L., Pearce, J., Milner, J., Mackenzie, I. A., Doherty, R. M. & Wilkinson, P. 2017. Socioeconomic and urban-rural differentials in exposure to air pollution and mortality burden in England. *Environmental Health,* 16**,** 1-10.

Ministry of Housing Communities and Local Government. 2019. *The English Indices of Deprivation 2019: Technical report* [Online]. Available: <https://assets.publishing.service.gov.uk/government/uploads/system/uploads/attachment_data/file/833951/IoD2019_Technical_Report.pdf> [Accessed 12/04/2023].

Nomis. 2025. *Nomis: Official census and labour market statistics* [Online]. Available: <https://www.nomisweb.co.uk/> [Accessed 16/04/2025].

Norman, P., Lloyd, C., Mclennan, D., Ferguson, S. & Catney, G. 2024. 50-year deprivation trajectories: local area change in England and Wales, 1971–2021. *Applied Spatial Analysis and Policy***,** 1-26.

Office for National Statistics. 2023. *Lower layer Super Output Areas (December 2021) EW Population Weighted Centroids* [Online]. Available: <https://geoportal.statistics.gov.uk/datasets/79fa1c80981b4e4eb218bbce1afc304b_0/explore?location=52.686943%2C-2.430938%2C7.37> [Accessed 14/10/2025].

Pugsley, K. L., Stedman, J. R., Brookes, D. M., Kent, A. J., Morris, R. J., Whiting, S. L., Wareham, J. V., Goodhand, A., Pepler, A., Thorp, T. M. & Gorji, S. 2023. *Technical report on UK supplementary modelling assessment under the Air Quality Standards Regulations 2010 for 2021* [Online]. Ricardo Energy & Environment. Available: <https://uk-air.defra.gov.uk/assets/documents/reports/cat09/2303151632_2021_PCM_technical_report.pdf> [Accessed 26/03/2024].
